# Comparative analysis of the cold acclimation and freezing tolerance capacities of seven diploid *Brachypodium distachyon* accessions

**DOI:** 10.1093/aob/mct283

**Published:** 2013-12-08

**Authors:** Katia Colton-Gagnon, Mohamed Ali Ali-Benali, Boris F. Mayer, Rachel Dionne, Annick Bertrand, Sonia Do Carmo, Jean-Benoit Charron

**Affiliations:** 1 McGill University, Department of Plant Science, 21,111 Lakeshore, Sainte-Anne-de-Bellevue, Canada; 2 Agriculture and Agri-food Canada, Soil and Crops Research and Development Centre, 2560 Hochelaga Blvd, Quebec, Canada; 3 McGill University, Department of Pharmacology and Therapeutics, 3655 Promenade Sir-William-Osler, Montreal, Canada

**Keywords:** *Brachypodium distachyon*, cold acclimation, *COR*413, flowering, freezing tolerance, ice recrystallization inhibition, fructans, phenological development, proline, vernalization, *VRN1*, winter hardiness

## Abstract

**Background and Aims:**

Cold is a major constraint for cereal cultivation under temperate climates. Winter-hardy plants interpret seasonal changes and can acquire the ability to resist sub-zero temperatures. This cold acclimation process is associated with physiological, biochemical and molecular alterations in cereals. *Brachypodium distachyon* is considered a powerful model system to study the response of temperate cereals to adverse environmental conditions. To date, little is known about the cold acclimation and freezing tolerance capacities of *Brachypodium*. The main objective of this study was to evaluate the cold hardiness of seven diploid *Brachypodium* accessions.

**Methods:**

An integrated approach, involving monitoring of phenological indicators along with expression profiling of the major vernalization regulator *VRN1* orthologue, was followed. In parallel, soluble sugars and proline contents were determined along with expression profiles of two *COR* genes in plants exposed to low temperatures. Finally, whole-plant freezing tests were performed to evaluate the freezing tolerance capacity of *Brachypodium*.

**Key Results:**

Cold treatment accelerated the transition from the vegetative to the reproductive phase in all diploid *Brachypodium* accessions tested. In addition, low temperature exposure triggered the gradual accumulation of *BradiVRN1* transcripts in all accessions tested. These accessions exhibited a clear cold acclimation response by progressively accumulating proline, sugars and *COR* gene transcripts. However, whole-plant freezing tests revealed that these seven diploid accessions only have a limited capacity to develop freezing tolerance when compared with winter varieties of temperate cereals such as wheat and barley. Furthermore, little difference in terms of survival was observed among the accessions tested despite their previous classification as either spring or winter genotypes.

**Conclusions:**

This study is the first to characterize the freezing tolerance capacities of *B. distachyon* and provides strong evidence that some diploid accessions such as Bd21 have a facultative growth habit.

## INTRODUCTION

Cold is one of the major constraints restricting cereal cultivation under temperate climates (Kosova *et al.*, 2011). Freezing temperatures encountered late in spring, early in autumn and during winter severely alter plant survival and thus represent a significant impediment for yield improvement. To circumvent some of the limitations imposed by freezing temperatures, hardy cereals efficiently interpret and respond in a co-ordinated manner to environmental cues that signal seasonal changes. As a consequence, winter genotypes can acquire the ability to resist freezing temperatures, whereas spring genotypes will remain limited in their ability to resist freezing (Limin *et al.*, 2007). Freezing tolerance in plants is thus not a constitutive trait and is expressed following exposure to low non-freezing temperatures, a process known as cold acclimation (Guy, 1990; Thomashow, 1999). Numerous studies have characterized in depth the cold acclimation process in cereals and other plants such as *Arabidopsis thaliana* and have established that this process is associated with numerous physiological, biochemical and molecular alterations (Houde *et al.*, 1992; Danyluk *et al.*, 1994; Crosatti *et al.*, 1996; Limin *et al.*, 1997; Stockinger *et al.*, 1997; Grossi *et al.*, 1998; Jaglo-Ottosen *et al.*, 1998; Thomashow, 1999; NDong *et al.*, 2002; Vogel *et al.*, 2005; Van Buskirk and Thomashow, 2006; Badawi *et al.*, 2007; Galiba *et al.*, 2009). These alterations are regulated by a complex multigenic system that revolves around the induced expression of several cold-regulated (*COR*) genes (Thomashow, 2001). This transcriptional response is thus essential for the development of freezing tolerance and will ultimately promote self-adaptive features, such as increased levels of carbohydrates, soluble proteins, proline and organic acids, photosynthetic adjustments, appearance of new enzyme isoforms, and modifications in the lipid membrane composition, necessary for the plant to protect critical cell structures and vital physiological processes (Hughes and Dunn, 1996; Hüner *et al.*, 1998; McNeil *et al.*, 1999; Thomashow, 1999).

Despite these self-adaptive features, sub-zero temperatures remain fatal to the sensitive floral meristems. Hardy cereals thus have a vernalization requirement that delays the transition from the vegetative to the reproductive phase. Exposure to a period of low non-freezing temperatures modulates the expression of specialized vernalization genes, which in turn promotes flowering only in the milder conditions of spring. Vernalization is thus initiated within the same time frame as the induction of the cold acclimation pathway. Despite this temporal overlap, no direct molecular link has been clearly established between vernalization and the cold acclimation processes. Nonetheless, several studies have pointed out that the characterization of the vernalization requirements of a cereal provides key information about its freezing tolerance capacity by showing that the transition from the vegetative to the reproductive phase is associated with the downregulation of *COR* genes and the upregulation of the vernalization gene *VRN-1* (Fowler *et al.*, 1996*a*, *b*; Mahfoozi *et al.*, 2001*a*, *b*; Dhillon *et al.*, 2010). As a result, full expression of cold hardiness genes only occurs in the vegetative phase, and plants in the reproductive phase have a limited ability to accumulate *COR* gene transcripts and to cold acclimate. In addition, plants that are still in the vegetative phase have the ability to re-acclimate even after periods of exposure to warm temperatures, whereas plants in the reproductive phase only have a poor ability to re-acclimate (Mahfoozi *et al.*, 2001*a*).

In the context of increasing demand on crops for food and biofuel production, breeding for cold tolerance holds promises for enhanced yield and extended cultivation seasons. It is thus important to gain in-depth knowledge about the cold response mechanisms of cereal plants and the intricate molecular circuitry involved. In order to support research initiatives, it is crucial to have a model plant which, in addition to possessing a small physical size, rapid life cycle and undemanding growth requirements, can make it amenable to perform high-throughput screening routines, functional analyses and transformation procedures (Draper *et al.*, 2001).

With its status of a major staple food and its compact genome of 441 Mbp (Bennett *et al.*, 2000), rice (*Oryza sativa*) was initially proposed as such a model for cereal plants. However, its use as a model organism for temperate cereals and forages has remained a matter of debate despite extensive international efforts that have led to the development of comprehensive genetic maps and imposing expressed sequence tag (EST) and germplasm collections. Nevertheless, the lack of microsynteny conservation, the presence of multiple small rearrangements and the fact that rice does not necessarily exhibit all the traits relevant to temperate cereal crops such as freezing tolerance and vernalization have considerably hampered the initial interest in this plant (Draper *et al.*, 2001; Faricelli *et al.*, 2010).

*Brachypodium distachyon* is an annual temperate wild grass that originates from Mediterranean and Middle East countries where sub-zero temperatures are frequently observed (Opanowicz *et al.*, 2008; Vogel *et al.*, 2010). It has many appealing biological attributes including self-fertility, small stature, short generation time and efficient transformation (Garvin, 2007; Alves *et al.*, 2009; Vogel and Bragg, 2009). Moreover, *Brachypodium* has a small sequenced genome (272 Mbp), and spring and winter diploid accessions have been classified according to the capacity to flower with or without cold exposure (Vogel *et al.*, 2010). There is strong chromosomal synteny between *Brachypodium* and other temperate cereals, and about 77 % of the genes retrieve significant matches in *Triticeae* EST databases (Huo *et al.*, 2009). This body of evidence has led researchers to propose *Brachypodium* as an appropriate model to study the response of temperate cereals to their environment. As a result, the *Brachypodium* model has proved its value in a number of biotic and abiotic stress tolerance studies (Schwartz *et al.*, 2010; Luo *et al.*, 2011; Peraldi *et al.*, 2011), clearly demonstrating its potential for understanding and ultimately improving abiotic stress tolerance in temperate cereals.

To date, little is known about the capacities of *Brachypodium* to cold acclimate and develop freezing tolerance. A recent study by Li *et al.* (2012) has demonstrated that *Brachypodium* has the molecular circuitry necessary to activate *COR* gene expression. Despite this leap forward, the extent of *Brachypodium*'s capacity to resist freezing is still unknown. In this study, we investigated the cold hardiness capacity of *Brachypodium.* To achieve this goal, an integrated approach involving the monitoring of double-ridge (DR) formation and final leaf number (FLN) was used to verify the growth habit classification of seven diploid *Brachypodium* accessions. In addition, the cellular concentration of soluble sugars and proline were determined, along with the transcript accumulation profiles of orthologues of the major vernalization regulator *VRN1* and two *COR* genes at different stages of cold acclimation. Finally, whole-plant freezing tests (WPFTs) were performed in order to characterize fully the freezing tolerance capacity of *Brachypodium*. This study is thus critical to evaluate the potential contribution of *B. distachyon* to cold hardiness research.

## MATERIALS AND METHODS

### Plant material and growth conditions

Seeds of *Brachypodium distachyon* spring accessions Bd2-3, Bd3-1, Bd21 and Bd30-1, and winter accessions Bd1-1, Bd18-1 and Bd29-1 were soaked for 2 h in sterile distilled water at room temperature, after which the lemma was removed. The seeds were then sterilized in 70 % ethanol, rinsed with sterile distilled water and sterilized again in 1·3 % sodium hypochlorite solution according to Vain *et al.* (2008) and Alves *et al.* (2009). The seeds were placed between two sterile filter papers imbibed with sterile distilled water in a Petri dish at 4 °C in the dark for 1 week. This stratification treatment is essential for the synchronization of germination of all *Brachypodium* accessions. Seeds were sown in pots containing Agro Mix^®^ (Plant Products Co. Ltd) and grown until the three-leaf stage (approx. 10 d) at 20 °C with a 16 h photoperiod and a photosynthetic photon flux density of 150 µmol m^−2^ s^−1^. At the end of this period, control non-acclimated plants were harvested (NA0) or maintained under the same light and temperature conditions for 5 (NA5), 7 (NA7), 21 (NA21) and 45 d (NA45) to provide adequate controls for the different cold acclimation (CA) time points. Cold acclimation was performed by subjecting plants at the three-leaf stage to a temperature of 4 ± 1 °C under either an 8 h photoperiod [short day (SD)] or a 16 h photoperiod [long day (LD)] at a photosynthetic photon flux density of 150 µmol m^−2^ s^−1^ for different periods of time as specified for each experiment. Deacclimated plants (DA1) were exposed to cold for 28 d (sugar and proline assays) or 42 d [quantitative real-time PCR (qPCR)] and returned to 20 °C under a 16 h photoperiod for 1 d. All plant samples were collected at the same time of day, 4 h after the beginning of the light period.

### Double-ridge formation

A dissection of the crown and an analysis of the shoot apex development identified the DR stage (Kirby and Appleyard, 1987; Kirby, 1990). Three-leaf stage plants were maintained under control conditions or exposed to 4 °C for the specified periods of time. After each treatment, plants were returned to 20 °C under a 16 h photoperiod until DR formation. An average of ten plants per accession were dissected at each of the ten low temperature (LT) treatments, and the mean number of days required to achieve DR formation was recorded to determine the influence of the LT treatment on the rate of phenological development. The experiment was repeated three times with independent biological replicates.

### Final leaf number measurements

Leaves on the main shoot were numbered and the plants were grown until the flag leaf emerged and the FLN could be determined (Wang *et al.*, 1995). Vernalization saturation was reached once the LT treatment no longer reduced the FLN. A minimum of ten plants was used at each of the ten LT treatments under both SD and LD conditions. The experiment was repeated three times with independent biological replicates.

### Whole-plant freezing test

For the WPFTs, 21 seedlings (three of each of the seven *Brachypodium* accessions tested) were grown until the three-leaf stage in a circular pattern in 4 inch round pots at 20 °C. Plants were either kept under the same conditions for an additional 5 d (NA5 plants) or cold acclimated at 4 °C for 28 d (CA28). Following acclimation, WPFTs were performed in a programmable Percival low-temperature chamber (LT-36VL) specifically designed to measure cold hardiness in plants. After a 4 h equilibration period at –2 °C during which plants were seeded with ice chips to initiate freezing (Charron *et al.*, 2008), the temperature was lowered by 1 °C h^−1^, the temperature being decreased in the first 5 min followed by a 55 min plateau. Plants were tested between –7 and –12 °C. At the end of each temperature plateau, three randomly selected pots of NA and CA plants were withdrawn from the chambers for a total of six pots per plateau. To minimize light stress effects after the freezing treatment, plants were thawed at 4 °C for 24 h in the dark before returning to normal growth conditions. After 2 weeks, survival counts were taken and the 50 % lethal temperature (LT_50_) calculated. A total of nine plants per accession, per treatment, per plateau, per experiment were removed from the incubator. The experiment was repeated four times with independent biological replicates.

### Proline and water-soluble sugar assays

For free proline and water-soluble sugar (WSS) content assays, the aerial parts of nine plants for each accession were used at each of the seven LT treatments. The experiment was repeated three times with nine independent biological replicates.

Free proline content was analysed using a colorimetric assay (Ábrahám *et al.*, 2010). Proline was extracted by grinding 100 mg of dried plant material with 500 µL of 3 % (w/v) sulfosalicylic acid. The extract was centrifuged at 13 000 rpm for 5 min at room temperature. A 0·1 mL aliquot of the extract was mixed with 0·5 mL of a solution of acidic ninhydrin [40 % acidic ninhydrin (8·8 µm nihydrin, 10·5 m glacial acetic acid, 2·4 m orthophosphoric acid), 40 % glacial acetic acid and 20 % of 3 % sulfosalicylic acid]. The samples were incubated for 60 min at 96 °C and the reaction was terminated by incubating the samples on ice for 5 min. The samples were then extracted by adding 1 mL of toluene and vortexing for 20 s. The absorbance at 520 nm was measured using toluene as a reference. The standard curve was made using l-proline in a range of 0–57·5 µg mL^−1^. Free proline content [μmol g^−1^ fresh weight (f. wt)] was calculated according to Bates *et al.* (1973):

(1) $$\eqalign{{\rm Free \ proline}= \, ({\rm \mu g} \ {\rm proline} \ {\rm mL}^{-1}\times {\rm mL} \ {\rm toluene}) \cr / 115 \! \cdot \! 5\,{\rm \mu g} \ {\rm\mu mol}^{-1}/({\rm g} \ {\rm sample}/5)}$$

Total WSS content was assessed according to Luo *et al.* (2011) with modifications to allow the data to be collected using a microplate reader (Galicia *et al.*, 2009). Total WSS was extracted from 5 mg of plant dry tissues with 1 mL of distilled water by vortexing. The extract was incubated at 70 °C for 45 min, vortexed every 15 min and centrifuged at 13 000 rpm for 20 min at room temperature. The supernatant was diluted 1:10 and 50 µL of this sample was mixed with 100 µL of anthrone solution [100 mg of anthrone in 100 mL of 95 % (w/w) H_2_SO_4_] in a 96-well microplate. The microplates were shaken at 150 rpm for 10 min at room temperature and incubated at 100 °C for 4 min. After cooling, the absorbance at 630 nm was measured. The standard curve was made using sucrose in a range of 0–200 µg mL^−1^. The WSS content in mg of sucrose g^−1^ of dry plant material was calculated as:

(2) $$ \eqalignno{{\rm WSS}= \, ({\rm mg \ of \ sucrose/mg \ of \ starting \ dry \ plant \ material}) \cr \times {\rm dilution \ factor}}$$

### Quantification of fructans

Dried ground material (100–200 mg) was incubated in 6 mL of deionized H_2_O at 80 °C for 20 min. The extracts were then incubated overnight at 4 °C and were subsequently centrifuged for 10 min at 1500 *g*. A 1 mL sub-sample of the supernatant was collected for quantification of fructans. High degree of polymerization (HDP, from DP 15 to DP 200) fructans and LDP (DP 10) fructans were analysed using a Waters HPLC analytical system controlled by the Empower II software. Samples were kept at 4 °C throughout the analysis within the Waters 717^plus^ autosampler. The HDP fructans were separated on a Shodex KS-804 column preceded by a Shodex KS-G pre-column (Shodex, Tokyo, Japan) eluted isocratically at 50 °C with deionized water at a flow rate of 1·0 mL min^−1^, and were detected on a refractive index detector (Model 2410, Waters). The DP of HDP fructans was estimated by reference to a standard curve established with seven polymaltotriose pullulan standards (Shodex Standard P-82) ranging from a molecular weight of 0·58 × 10^4^ to 85·3 × 10^4^. The retention time on the Shodex column is a function of the log of the molecular weight of pullulan molecules. The concentrations of both HDP and LDP fructans are expressed on an equivalent fructose basis.

### RNA extraction, cDNA synthesis and quantitative real-time PCR analysis

Total RNA was isolated from the aerial parts of plants (*n* = 3 plants per time point) using the RNeasy plant mini kit (Qiagen). All RNAs were treated with DNase I (Qiagen) to remove genomic DNA. RNA integrity was visually assessed with agarose gel electrophoresis. A 2 µg aliquot of total RNA was then reverse transcribed using the AffinityScript QPCR cDNA Synthesis Kit (Agilent Technologies) according to the manufacturer's recommendations. Parallel reactions were run for each RNA sample in the absence of AffinityScript reverse transcriptase (no reverse transcriptase control) to assess any genomic DNA contamination. The cDNA products were diluted in water to 400 ng μL^−1^ and stored at –20 °C.

Quantitative PCR assays were conducted in triplicate in an Mx3000 real-time thermal cycler (Agilent Technologies) with Brilliant III Ultra-Fast SYBR^®^ Green QPCR master mix (Agilent Technologies). First-strand cDNA was used as template for PCR amplification using gene-specific primers (Table 1). Amplification of the 18S rRNA gene was used as an internal standard. Amplification was performed in a 15 µL reaction containing 1× SYBR Green master mix, 350 nm of each primer, 30 nm reference dye ROX and 80 ng of cDNA template. The PCR thermal cycling parameters were 95 °C for 2 min followed by 40 cycles of 95 °C for 5 s and 60 °C for 20 s. Three technical replicates were used, and the experiment was repeated three times with different biological replicates. Controls without template were included for all primer pairs.

**Table 1.** List of primers used in this study

Primer name	Primer sequence (5′ to 3′)	Amplicon size (bp)
BradiVRN1-F	5′-CAGATCCAGAAAGAACCAGCTAA-3′	220
BradiVRN1-R	5′-GCGATTACTGATATTTGTTGTTGG-3′	
BradiCOR413-F	5′-AGGTTGGTTGCTGGATTGCGTTC-3′	76
BradiCOR413-R	5′-TCCAGCCAATCAGGAAAGTGGCG-3′	
BradiIRI-F	5′-AACTGGCAACAACAACGCCGTG-3′	147
BradiIRI-R	5′-ACGATGTGGTTGCTCCCGGATAC-3′	
Bradi18S-F	5′-GAAGTTTGAGGCAATAACAGGTCT-3′	131
Bradi18S-R	5′-ATCACGATGAATTTCCCAAGATTAC-3′	

Raw fluorescent data (background-subtracted data) provided by the Mx-Pro QPCR software (Agilent Technologies) were analysed using the PCR Miner program (http://miner.ewindup.info/miner; Zhao and Fernald, 2005). For each LT treatment, data were expressed as a ratio of gene of interest expression to 18S expression.

### Statistical analysis

Unless stated otherwise, all the experiments were carried out three times in a complete randomized design with a minimum of three replicates. All data were subjected to an analysis of variance (ANOVA) using the general linear model (GLM) procedure in SAS (SAS Institute, Cary, NC, USA) to identify significant treatment effects. Comparisons between means were made using least significant differences (LSDs) at a 0·05 probability level when ANOVA indicated model and treatment significances.

## RESULTS

### Phenological development

**Fig. 1.**
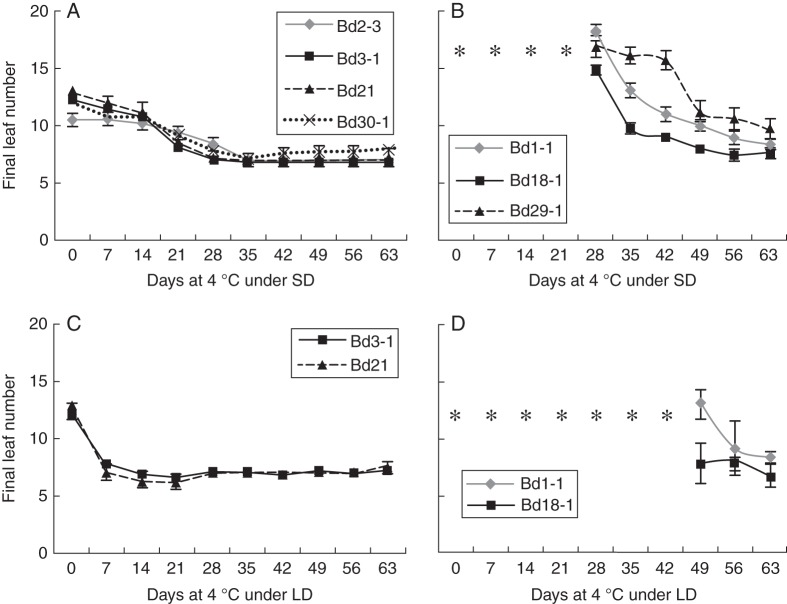
Final leaf number of seven diploid *Brachypodium distachyon* accessions in response to cold exposure. Final leaf number was compiled for *Brachypodium* accessions previously shown to have spring (left panel) or winter (right panel) growth habits when cold acclimated under (A and B) SD and (C and D) LD conditions. Time points where plants did not flower during the course of the experiment are indicated by an asterisk. Statistically significant differences (*P* < 0·05) in final leaf number were observed until 28 d for Bd3-1, Bd21 and Bd30-1, until 35 d for Bd2-3, until 49 d for Bd29-1 and until 56 d for accessions Bd1-1 and Bd18-1.

Molecular and physiological studies aimed at understanding the mechanism of freezing tolerance in cereals have revealed that this process is closely associated with the vernalization response (Fowler *et al.*, 1996*a*; Danyluk *et al.*, 2003; Dhillon *et al.*, 2010). Thus, with the goal of unravelling the cold acclimation pattern of seven *Brachypodium* accessions, we initially opted for a classical approach by determining the effects of low non-freezing temperatures on their phenological development. As a first step, we tested the effects of cold exposure on the FLN of different accessions under SD and LD conditions. In all *Brachypodium* accessions tested in the current study, exposure to cold and SD conditions had noticeable effects on their FLN. The FLN of the four accessions able to flower without being exposed to LT clearly decreased following the cold treatment under SD conditions (Fig. 1A). Their FLN declined from 11–13 leaves under normal conditions to 7–8 leaves after 28 d of cold treatment. Exposure to cold for periods >28 d did not further reduce the FLNs of accessions Bd3-1, Bd21 and Bd30-1.

The three winter accessions did not transition from the vegetative to the reproductive phase in the time frame of the experiment unless they were cold treated under SD conditions for a minimum of 28 d. At this time point, these accessions showed elevated FLN (15–18 leaves), and a steady decrease in the FLN was observed during the cold treatment until no noticeable changes (7–10 leaves) were observed for exposure longer than 49–56 d (Fig. 1B).

We initially chose to perform cold treatment under SDs as this experimental set-up resembles the natural conditions observed during the autumn and is known to promote strong cold acclimation responses in temperate cereals (Fowler *et al.*, 2001). However, this experimental design introduces an obvious confounding factor by simultaneously varying the temperature and the photoperiod. Thus, to determine the respective contributions of LT and daylength reduction to the FLN changes observed in Fig. 1A and B, we monitored fluctuations in the FLNs of *Brachypodium* accessions cold treated under LD conditions. Under these conditions, we observed a significant FLN reduction in two *Brachypodium* accessions able to flower without being exposed to LT (Bd3-1 and Bd21; Fig. 1C). The FLNs of these accessions declined, respectively, from 11 and 13 leaves under normal conditions to six and seven leaves after only 14 d of cold treatment. Interestingly, LD conditions significantly delayed the flowering transition of winter accessions since these accessions only formed spikes after spending a minimum of 49 d at 4 °C. At this time point, the winter accessions tested (Bd1-1 and Bd18-1) had FLNs of eight and 13, respectively, and prolonged LT exposure up to 63 d further reduced their FLNs to seven and eight (Fig. 1D). Together, the results presented in Fig. 1 demonstrate that a LT treatment reduces the FLN of *Brachypodium* accessions in both SD and LD conditions and that the rate of this reduction is photoperiod dependent.

**Fig. 2.**
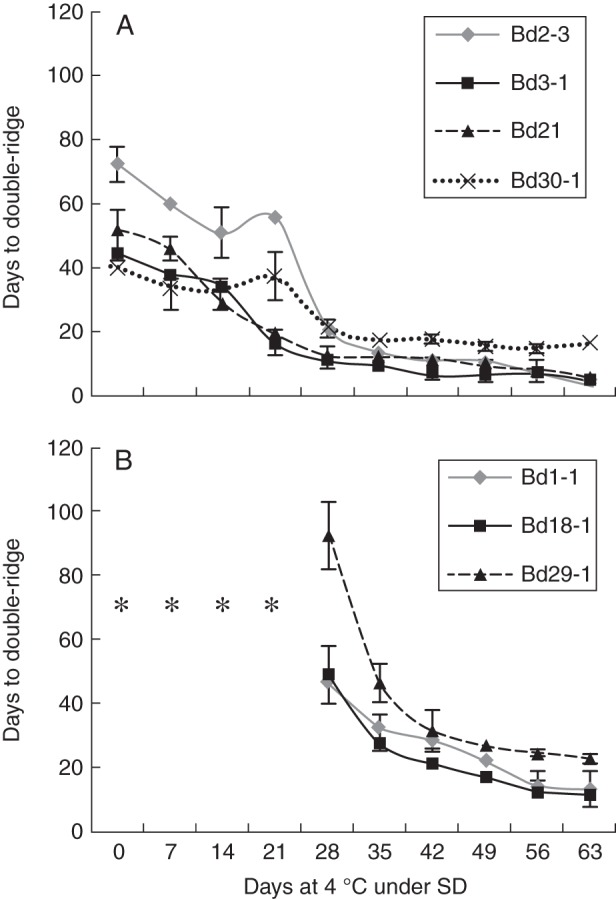
Apical development of seven diploid *Brachypodium distachyon* accessions exposed to cold (4 °C) for the indicated time. Double-ridge formation was compiled for *Brachypodium* accessions previously shown to have (A) spring or (B) winter growth habits. Time points where plants never formed the double-ridge structure during the course of the experiment are indicated by an asterisk. Statistically significant differences (*P* < 0·05) in days to double-ridge values were observed until 28 d for Bd21 and Bd 30-1, until 42 d for Bd2-3 and Bd 3-1 and until 49 d of treatment for Bd1-1, Bd18-1 and Bd29-1.

In order to expand our phenological characterization, we monitored at different stages of cold exposure under SDs the formation of the DR structure, a well-known indicator of the transition from the vegetative to the reproductive stages in grasses (Kirby, 2002). As expected, dissection of the shoot apices established that four of the accessions, Bd2-3, Bd3-1, Bd21 and Bd30-1, entered the reproductive phase when grown at 20 °C for their complete life cycle. This observation confirms that those accessions do not require a cold treatment in order to flower (point 0 day; Fig. 2A). The days of growth required until DR formation (from the end of the vernalization treatment to the DR formation) varied markedly among those accessions, clearly indicating natural genetic variation. Indeed, accession Bd2-3 needed the most days to reach the DR stage, in contrast to accessions Bd3-1, Bd21 and Bd30-1 that presented very similar profiles when maintained under control conditions (point 0 day; Fig. 2A). Interestingly, a cold treatment under SD conditions reduced the numbers of days required until DR formation following LT treatment in these four accessions (Fig. 2A).

Contrasting results were obtained with the three other accessions tested (Bd1-1, Bd18-1 and Bd29-1; Fig. 2B). Under SD conditions, these accessions only formed the DR structure after spending a minimum of 28 d at 4 °C, indicating that unlike accessions Bd2-3, Bd3-1, Bd21 and Bd30-1 (Fig. 2A), they have a strong vernalization requirement and are true winter accessions. For these accessions, vernalization saturation was reached after a cold treatment of 49 d (Fig. 2B).

The results presented in Fig. 2 also indicate that a cold treatment under SD conditions can promote the formation of the DR structure earlier in the development of most *Brachypodium* accessions with no absolute vernalization requirement. Indeed, by adding the number of days of growth before the vernalization treatment (14 d), the duration of the vernalization treatment at a given time point and the corresponding number of days needed to observe the DR structure once the plants are returned to normal conditions, it is possible to estimate whether an LT treatment under SD conditions can stimulate early flowering in *Brachypodium* plants. In comparison with untreated plants, 21 d of exposure to LT and SD conditions accelerated the DR formation in accessions Bd3-1 and Bd21 by 6 and 11 d, respectively, while 28 and 35 d exposures accelerated the flowering transition of accession Bd3-1 by 23 and 24 d. On the other hand, accession Bd30-1 did not develop the DR structure earlier following the nine LT treatments.

### Molecular response to low temperatures

In order to substantiate our phenological development observations and further to highlight the link between the vernalization and the cold acclimation responses in *Brachypodium*, we opted to juxtapose the expression profiles of orthologues of the vernalization regulator *VRN1* and of two *COR* genes already shown to be differentially expressed in response to cold in monocots (Breton *et al.*, 2003; Tremblay *et al.*, 2005; Li *et al.*, 2012).

In agreement with Higgins *et al.* (2010), *Bradi1g08340·1*, hereafter named *BradiVRN1*, was selected for this study because of its high level of sequence homology to the wheat *VRN1* (Supplementary Data Fig. S1). The protein encoded by *BradiVRN1* shares high homology with *Triticum monococcum VRN1* (Pidal *et al.*, 2009) (89 % identity and 92 % similarity) and *Triticum aestivum VRT-1* (Danyluk *et al.*, 2003) (84 % identity and 89 % similarity; Fig. S1). Like most MADS box transcription factors, the protein encoded by *BradiVRN1* contains the usual three conserved regions (MADS, I and K domains) and a C-terminal domain. As expected, lower sequence similarity is observed in the C-terminal region since this region was shown to diverge substantially between MADS box orthologues (Theissen *et al.*, 1996). Sequence analysis further revealed that *BradiVRN1* possesses a bipartite nuclear targeting domain that is conserved in all the MADS box transcription factors as well as conserved phosphorylation sites in the C-terminal region (Fig. S1; Danyluk *et al.*, 2003).

**Fig. 3.**
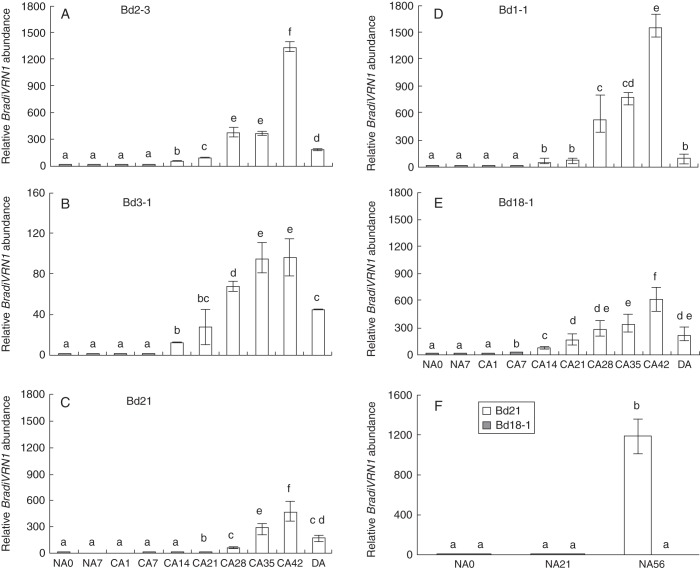
Expression analysis of *BradiVRN1* in response to low temperatures. (A–C) Relative transcript accumulation of *BradiVRN1* in *Brachypodium* accessions Bd2-3, Bd3-1 and Bd21. (D and E) Relative transcript accumulation of *BradiVRN1* in winter *Brachypodium* accessions Bd1-1 and Bd18-1. (F) Relative transcript accumulation of *BradiVRN1* in Bd21 and Bd18-1 plants not exposed to 4 °C. Letters above bars represent statistical significance (*P* < 0·05); different letters indicate statistically different fold expression.

*Brachypodium* accessions Bd1-1, Bd2-3, Bd3-1, Bd18-1 and Bd21 were selected to highlight variations in transcript accumulation of *BradiVRN1* in response to cold exposure because of their contrasting differences in phenological development. Non-acclimated (NA0 and NA7) plants and plants cold acclimated for 1 and 7 d (CA1 and CA7) showed minimal *BradiVRN1* transcript accumulation, whereas progressively longer cold exposure led to a gradual accumulation of the transcript in all accessions tested (Fig. 3A–E). Maximum accumulation was reached after 42 d, and transcript levels remained elevated after 1 d of deacclimation in all accessions (DA1; Fig. 3A–E). The fold change values in *BradiVRN1* transcripts varied greatly among accessions, with overall differences ranging from 100-fold (Bd3-1) to 1500-fold (Bd1-1) increases after 42 d of cold treatment. No obvious correlation was observed between the magnitude of the variations in *BradiVRN1* transcripts and the growth habits of the accessions tested (Fig. 3A–E). Interestingly, increased accumulation of *BradiVRN1* transcripts is also observed in ageing Bd21 plants never exposed to 4 °C, whereas the level of this transcript remains low and stable in ageing Bd18-1 plants (Fig. 3F).

**Fig. 4.**
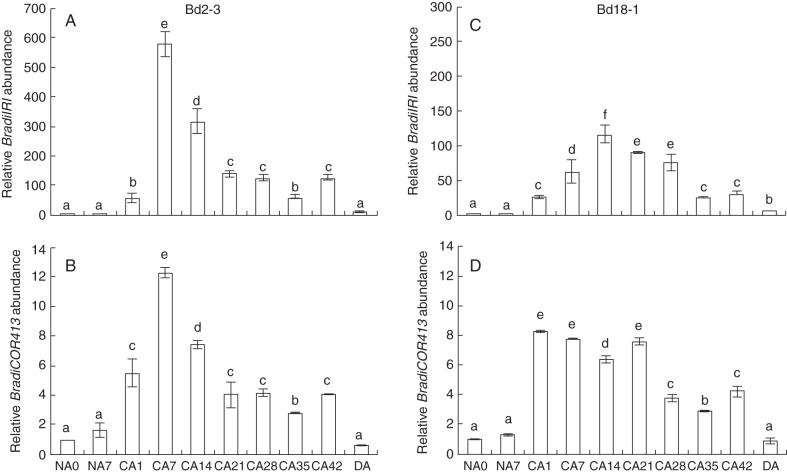
Accumulation of *COR* gene transcripts during cold acclimation in *Brachypodium distachyon*. Relative transcript accumulation of (A, B) *BradiIRI* and (C, D) *BradiCOR413* in Bd2-3 and Bd18-1 plants exposed to LT for up to 42 d. Relative transcript accumulation in above-ground tissues was measured by quantitative real-time PCR and normalized to 18S rRNA transcript levels. Letters above bars represent statistical significance (*P* < 0·05); different letters indicate statistically different fold expression.

Variations in the expression of *COR* genes can be correlated to the level of freezing tolerance in cereals (Houde *et al.*, 1992; Danyluk *et al.*, 1994; Fowler *et al.*, 1996*a*; Limin *et al.*, 1997; Danyluk *et al.*, 1998; Breton *et al.*, 2003). In this study, we thus explored the possibility of using *BradiIRI* (Bradi5g27350·1) and *Bradicor413* (Bradi1g07440·1) as potential markers for freezing tolerance in *Brachypodium*. As expected, the accumulation of *BradiIRI* increased following cold exposure in accessions Bd2-3 and Bd18-1 (Fig. 4A, C). In Bd2-3, maximum transcript accumulation was reached after 7 d at 4 °C, after which a steady decrease in accumulation was observed. In Bd18-1, the level of *BradiIRI* transcripts gradually increased until 14 d of cold exposure, after which a decrease in accumulation was also observed. The level of *BradiIRI* transcripts returned to control levels in both accessions after 1 d of deacclimation (Fig. 4A, C).

The *cold-regulated 413* gene was first identified in wheat and arabidopsis where the accumulation of its transcripts was correlated with the capacity of the plant to develop freezing tolerance (Breton *et al.*, 2003). Sequence homology searches allowed us to identify *Bradi1g07440·1* as the *Brachypodium* orthologue of *COR413* (Supplementary Data Fig. S2). A clear accumulation of *BradiCOR413* transcripts was observed after 1 d of cold treatment in both accessions tested (Fig. 4B, D). In Bd2-3, the maximum accumulation was observed after 7 d of cold exposure. Following that point, a gradual decrease in transcript abundance was observed until 21 d of cold treatment, after which the levels remained constant until the end of the cold treatment (Fig. 4B, D). In Bd18-1, *BradiCOR413* transcripts levels remained high during the first 3 weeks of cold treatment, after which a slight decrease in transcript levels was noticed (Fig. 4B, D). Upon deacclimation, *BradiCOR413* levels returned to control levels in both accessions (Fig. 4).

### Freezing tolerance determination

**Fig. 5.**
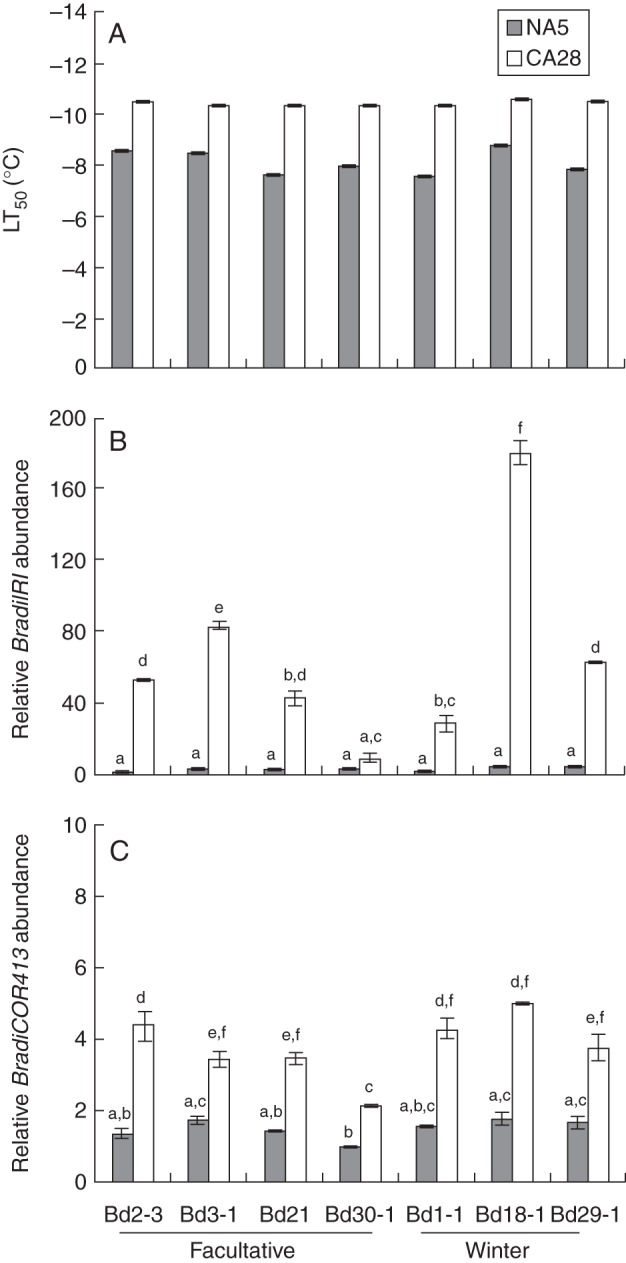
Freezing temperature tolerance (LT_50_) of seven diploid *Brachypodium distachyon* accessions. (A) Plants at the three-leaf stage were either kept under normal conditions (non-acclimated) for 5 d (NA5) or cold acclimated for 28 d (CA28) before being subjected to WPFTs. After 2 weeks in normal growth conditions, survival counts were taken and the LT_50_ was calculated. The experiment was repeated four times, and error bars indicate the standard deviation of the mean. (B and C) Relative transcript abundance of *BradiIRI* (B) and *BradiCOR413* (C) in NA5 and CA28 plants used for WPFTs of seven diploid *Brachypodium* accessions. Letters above bars represent statistical significance (*P* < 0·05); different letters indicate statistically different fold expression.

In order to determine the freezing tolerance capacities of the seven diploid *Brachypodium* accessions used in this study, WPFTs were conducted with plants grown under normal conditions (NA5) and plants that were cold acclimated for 28 d (CA28; Fig. 5A). The 28 d time point was selected based on our phenological development observations and gene accumulation data (Figs 1–4), as well as on reported distinctive variation in freezing tolerance between spring and winter wheat varieties observed after 4 weeks of cold exposure (Fowler and Limin, 2004). Furthermore, preliminary tests performed with control plants and plants exposed to cold for 7 and 14 d did not reveal significant differences in freezing tolerance between NA and CA *Brachypodium* plants (data not shown). Prior to WPFTs, no noticeable morphological differences that could explain variations in freezing tolerance were apparent between NA5 and CA28 *Brachypodium* plants. Following WPFTs, plants that did not survive the procedure were heavily damaged and evidently dead. A survival rate of 50 % was obtained for NA5 plants at temperatures ranging from –7·6 °C (Bd21) to –8·8 °C (Bd18-1). A 28 d period of cold exposure allowed an approx. 2 °C decrease in the LT_50_ values for all accessions, with survival rates of 50 % observed at temperatures ranging from –10·3 °C (Bd3-1) to –10·6 °C (Bd18-1). Overall, no obvious difference in freezing tolerance was observed between the group of accessions that do not require vernalization to flower (Bd2-3, Bd3-1, Bd21 and Bd30-1) and the winter type accessions (Bd1-1, Bd18-1 and Bd29-1; Fig. 5A).

The observed homogeneity in freezing tolerance levels between the seven *Brachypodium* accessions tested suggests similar *COR* gene transcripts levels after 28 d of cold acclimation. The accumulation levels of *BradiIRI* and *BradiCOR413* transcripts were thus monitored in NA5 and CA28 samples taken prior to the WPFTs. The accumulation levels of *BradiIRI* transcripts did not reflect the WPFT observations since pronounced variations in *BradiIRI* transcript levels were observed among the different accessions (Fig. 5B). After 28 d of cold acclimation, the strongest accumulation of *BradiIRI* transcripts was detected in Bd18-1, while 20 times fewer transcripts were detected in Bd3-1. On the other hand, *BradiCOR413* transcript accumulation was relatively stable among CA28 plants of all accessions, which better reflects their homogenous freezing tolerance capacities (Fig. 5C).

### Metabolic adjustments in response to low temperatures

**Fig. 6.**
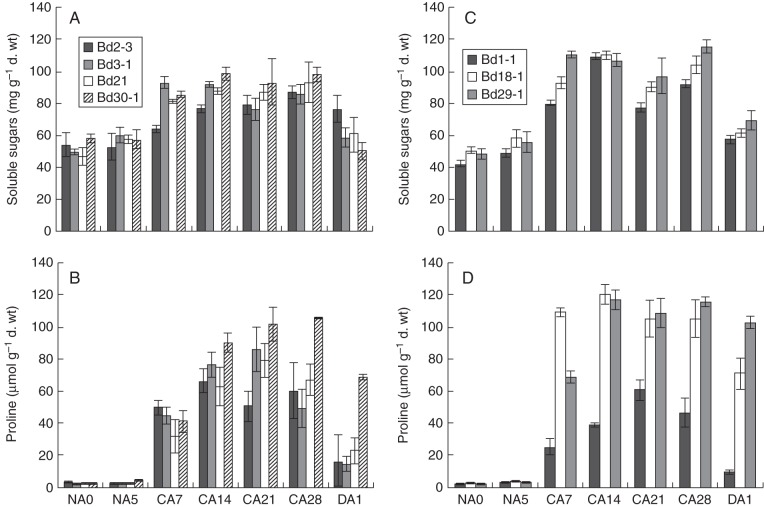
Water-soluble sugar and proline concentration in leaves of seven diploid *Brachypodium distachyon* accessions exposed to cold. (A and C) WSS and (B and D) proline concentration of non-acclimated plants (NA0 and NA5), cold-acclimated plants (CA7, CA14, CA21 and CA28) and de-acclimated plants (DA1) of accessions previously shown to have a spring growth habit (left) or winter growth habit (right). The error bars indicate standard deviation of the mean.

In order to characterize further the freezing tolerance of *Brachypodium*, we determined the effect of prolonged cold exposure on the content of WSS. Non-acclimated *Brachypodium* tissues contained minimum values of WSS, ranging from 43 µg mg^−1^ dry weight (d. wt) (Bd1-1) to 57 µg mg^−1^ d. wt (Bd30-1; Fig. 6A, C). A significant increase in the WSS concentration was noticed in all accessions when the plants were exposed to cold temperatures (Fig. 6A, C). After a 1 d de-acclimation period under normal growth conditions, a clear reduction of the WSS concentration was observed in all accessions (Fig. 6A, C). Interestingly, the WSS content was high in all *Brachypodium* accessions at the 28 d time point. However, these values varied greatly among accessions, which could suggest that a global assessment of WSS does not have the specificity needed to estimate precisely the freezing tolerance capacity of *Brachypodium* plants. To circumvent this limitation, we opted to measure fructans, a well-characterized group of storage carbohydrates known to accumulate during cold acclimation in temperate grasses (Livingston *et al.*, 2009). The chromatographic characterization of fructans revealed that both LDP and HDP fructans are present in above-ground tissues of *Brachypodium* plants (Table 2). In all accessions tested, LDP and HDP fructans were found to increase in response to LT treatment. In comparison with LDP fructans, higher concentrations of HDP fructans were observed in the seven *Brachypodium* accessions tested (Table 2). Furthermore, HDP fructan values in cold-acclimated plants did not show any significant variation among the different accessions, which suggest that this measurement could potentially be used as a freezing tolerance indicator for the *Brachypodium* model.

**TABLE 2.** Changes in LDP and HDP fructans in above-ground tissues of seven diploid *Brachypodium distachyon* accessions in response to cold acclimation

Treatment	Accession	Growth habit	LDP (mg g^−1^ d. wt)	HDP (mg g^−1^ d. wt)
NA5	Bd2-3	Facultative	0·16 ± 0·02^a^	1·05 ± 0·08^a^
	Bd3-1	Facultative	0·11 ± 0·03^a^	1·10 ± 0·10^a^
	Bd21	Facultative	0·06 ± 0·02^a^	1·12 ± 0·09^a^
	Bd30-1	Facultative	0·08 ± 0·02^a^	1·51 ± 0·13^a^
	Bd1-1	Winter	0·04 ± 0·02^a^	0·80 ± 0·06^a^
	Bd18-1	Winter	0·05 ± 0·01^a^	1·04 ± 0·04^a^
	Bd29-1	Winter	0·08 ± 0·01^a^	1·29 ± 0·05^a^
CA28	Bd2-3	Facultative	1·13 ± 0·09^e^	2·66 ± 0·21^ab^
	Bd3-1	Facultative	4·31 ± 0·22^f^	5·45 ± 0·62^bc^
	Bd21	Facultative	2·57 ± 0·11^d^	5·96 ± 2·90^bc^
	Bd30-1	Facultative	2·22 ± 0·11^c^	5·95 ± 0·43^bc^
	Bd1-1	Winter	1·99 ± 0·13^c^	7·55 ± 2·79^bc^
	Bd18-1	Winter	1·62 ± 0·05^b^	5·97 ± 0·09^bc^
	Bd29-1	Winter	1·38 ± 0·09^be^	8·74 ± 0·64^c^

Different letters indicate significant differences between means (*P* < 0·05).

We also wanted to determine if proline could be used as an indicator of the freezing tolerance level of *Brachypodium* accessions. Under control conditions, similar basal proline contents were observed in all accessions (Fig. 6B, D). As observed in the case of WSS, an elevated proline content was observed in all accessions throughout the cold treatment. Winter accessions Bd18-1 and Bd29-1 displayed the most robust proline profile throughout the cold treatment (Fig. 6B, D), whereas the lowest pool of proline was observed in winter accession Bd1-1. A decrease in proline concentration was observed in all accessions when plants were returned to normal conditions for 1 d (DA1). However, proline levels remained elevated in DA1 plants of accessions Bd18-1, Bd29-1 and Bd30-1, whereas levels returned close to control values in accession Bd1-1, Bd2-3, Bd3-1 and Bd21 (Fig. 6B, D).

## DISCUSSION

As part of our long-term objective of complete characterization of the freezing tolerance components of *Brachypodium distachyon*, we devised a classical approach involving the monitoring of phenological, molecular and metabolic indicators. Temperate cereal germplasms can be divided into three broad growth habit classifications – winter, facultative and spring. Winter varieties are freezing tolerant and require vernalization. In contrast, spring varieties barely tolerate freezing temperatures and do not require vernalization. The facultative growth habit lacks a clear definition and is considered by some as a sub-class of the winter growth habit. The facultative habit genotypes are usually freezing tolerant like winter varieties but lack vernalization requirements (von Zitzewitz *et al.*, 2005).

The *B. distachyon* diploid inbred lines used in this study were previously classified as either spring (Bd2-3, Bd3-1, Bd21 and Bd30-1) or winter genotypes (Bd1-1, Bd18-1 and Bd29-1) according to their capacity to flower or not without prior exposure to cold (Schwartz *et al.*, 2010). Our results, obtained from the monitoring of phenological indicators and the profiling of *BradiVRN1* transcript accumulation, corroborate the winter habit classification of accessions Bd1-1, Bd18-1 and Bd29-1. These accessions did not flower without being previously exposed to 4 °C for a minimum of 28 d under SD conditions (Figs. 1, 2). Non-acclimated winter accession plants were eventually grown for >200 d without any signs of flowering (data not shown), clearly highlighting their absolute vernalization requirement. The fact that sharp decreases in FLN and in the number of days to DR formation are observed for theses accessions is in agreement with reports on wheat and rye winter varieties (Limin *et al.*, 1996*b*). On the other hand, the trend observed for these two phenological indicators in *Brachypodium* ‘spring’ accessions did not exhibit the stable characteristic profiles generally observed in rye and wheat spring varieties (Fowler *et al.*, 1996*a*, *b*). A closer examination of the profiles obtained for the two phenological indicators used in this study revealed a closer match to facultative cereal varieties where a small but significant acceleration of the phenological development is usually observed when plants are exposed to cold. The acceleration in response to LT of the phenological development of accessions Bd2-3, Bd3-1, Bd21 and Bd30-1 observed during this study was unexpected, and implies that these accessions are facultative genotypes since this type of behaviour is uncommon for spring genotypes. At first sight, these results do not agree with the study of Schwartz *et al.* (2010) that reported no significant effect of vernalization (in terms of number of days) on the vegetative to reproductive phase transition in accessions Bd3-1, Bd21 and Bd30-1. However, this discrepancy can most probably be explained by the age difference of the plant material used in both studies. Indeed, Schwartz *et al.* (2010) used plants that were closer to the transition point before performing the vernalization treatment (4-week-old plants grown under a 20 h light/4 h dark photoperiod), wheras *Brachypodium* plants at the three-leaf stage (approx. 14 d old) were exposed to cold in the current study.

The genome of *Brachypodium* has been reported to contain multiple orthologues of the temperate cereal gene *VRN1*. On one hand, Schwartz *et al.* (2010) proposed that two genes (Bradi1g08340·1 and Bradi1g59250·1) could potentially be considered as *VRN1* orthologues, while another study clearly identified Bradi1g08340·1 as the only *VRN1* candidate in *Brachypodium* (Higgins *et al.*, 2010). For this study, Bradi1g08340·1 was selected based on its close sequence identity to wheat *VRN1*. Although further work is needed to confirm *BradiVRN1* (Bradi1g08340·1) as *VRN1*, the expression profiles presented in this study undoubtedly revealed several key characteristics that establish this gene as a vernalization gene (Yan *et al.*, 2003). The transcript accumulation patterns observed among accessions Bd1-1, Bd2-3, Bd3-1, Bd21 and Bd18-1 over a period of 42 d of cold exposure are consistent with profiles commonly observed in winter wheat varieties. These winter varieties generally express *VRN1* at low levels during the early stages of cold exposure, and progressively higher levels of this transcript can be detected until the 49–56 d mark, where a distinctive plateau is usually reached (Kane *et al.*, 2005). Furthermore, the abundance of this transcript remains elevated upon deacclimation after 42 d of cold treatment. While this expression pattern was expected for winter habit genotypes Bd1-1 and Bd18-1, it does not support the ‘spring habit’ classification of Bd2-3, Bd3-1 and Bd21 accessions. Spring cereal genotypes such as Manitou (wheat) and Morex (barley) constitutively express *VRN1* regardless of their vernalization status (Danyluk *et al.*, 2003; Kane *et al.*, 2005; von Zitzewitz *et al.*, 2005). Surprisingly, the transcript accumulation pattern of *BradiVRN1* in accessions Bd2-3, Bd3-1 and Bd21 (Fig. 3A, B, C) resembles the profiles observed in winter cereal varieties and is a very close match to the *VRN1* profile observed in the facultative barley variety Dicktoo (Danyluk *et al.*, 2003; Kane *et al.*, 2005; von Zitzewitz *et al.*, 2005). In addition, the present study demonstrates that LT is not necessarily the only determinant responsible for the increased accumulation of *VRN1* in facultative accessions, since this transcript accumulates at late developmental stages in accession Bd21 grown under control conditions (Fig. 3F). This observation, together with our DR and FLN data (Figs 1, 2), clearly suggests that accession Bd21 has a facultative growth habit.

The possibility that no spring habit *Brachypodium* accession can be found in the group of diploid accessions tested is further substantiated by our WPFT observations. Even if a cold acclimation period of 28 d only resulted in a modest 2 °C gain in freezing tolerance in *Brachypodium*, the fact that this gain is similarly observed among all accessions is another good indication that these accessions can be classified as facultative or winter habits. In addition, the LT_50_ values observed for non-acclimated plants are relatively low in comparison with values previously obtained for barley (approx. –2 °C; Limin *et al.*, 2007) and winter wheat varieties (approx. –4 °C; Fowler and Limin, 2004) grown under similar conditions. This suggests that *B. distachyon* has stronger constitutive freezing tolerance mechanisms compared with modern cultivated cereal varieties that rely on inducible mechanisms to develop freezing tolerance.

The *BradiIRI* and *BradiCOR413* transcript abundance analyses reported here provide additional evidence that some of the molecular mechanisms of freezing tolerance observed in hardy grasses are present in *Brachypodium*. Furthermore, the differential transcript accumulation patterns observed for both *COR* genes emphasizes the possibility that accessions Bd2-3, Bd3-1, Bd21 and Bd30-1 have facultative growth habits. Facultative genotypes, like winter types, are known to rely on induced *COR* gene expression to induce their freezing tolerance capacities but lack an absolute vernalization requirement (Karsai *et al.*, 2005). In addition, the decline observed in *BradiIRI* and *BradiCOR413* transcript accumulation in Bd2-3 and Bd18-1 (Fig. 4) is negatively correlated with the increase in *BradiVRN1* transcript levels (Fig. 3A, E). This suggests that *COR* gene transcripts become less abundant when the floral transition is about to be reached in these accessions. However, our results suggest that *BradiIRI* is not a reliable freezing tolerance indicator/marker for *Brachypodium*. This observation agrees well with results reported in Li *et al.* (2012), where 7-week-old cold-acclimated *Brachypodium* plants of accessions Bd1-1, Bd21-1 and Bd29-1 were shown to accumulate various levels of *BradiIRI* transcripts. On the other hand, *COR413* accumulation has been tightly correlated to the plant's capacity to develop freezing tolerance in wheat (Breton *et al.*, 2003), and our observations suggest that this gene could potentially be used as a freezing tolerance indicator for *Brachypodium* as well. Nevertheless, accessions or eventually transformants with clear phenotypic variations for freezing tolerance will have to be investigated either to support or to refute this assertion.

Several metabolites have been reported to give instantaneous snapshots of the physiology of a plant cell (Levitt, 1980). This is best exemplified by the existence of positive correlations between the level of freezing tolerance of cereals and their capacities to accumulate compatible solutes such as proline and WSS (Olien and Clark, 1993; Hurry *et al.*, 1995; Yoshida *et al.*, 1998; Vágújfalvi *et al.*, 1999). The distinctive difference in WSS concentration generally observed between spring and winter cereal genotypes was not observed among the *Brachypodium* diploid accessions tested. Again, this can probably be explained by the facultative behaviour displayed by accessions Bd2-3, Bd3-1, Bd21 and Bd30-1 throughout this study. Nonetheless, the lack of a clear trend among the WSS profiles observed in this study might just be a characteristic that sets apart the *Brachypodium* model, since Luo *et al.* (2011) did not find a distinctive difference in WSS among *Brachypodium* accessions even if clear differences in drought tolerance capacities were observed. However, our study provides evidence that the characterization of HDP fructans could serve as an alternative to the broad characterization of WSS in order to evaluate the freezing tolerance capacities of *Brachypodium* accessions. Our results revealed that *Brachypodium* accessions with similar freezing tolerance capacities accumulate HDP fructans at very comparable levels after 28 d of cold treatment. This finding does not seem to be limited to the *Brachypodium* model since similar correlations have been established in winter wheat, triticale and rye cultivars (Suzuki and Nass, 1988). However, while HDP fructans do accumulate in response to cold in *Brachypodium*, it is worth noting that the overall concentration values of HDP and LDP fructans remain low in comparison with values observed in cold-hardy grasses such as winter wheat varieties and annual bluegrass genotypes that can accumulate up to 100 mg g^−1^ d. wt under similar conditions (Suzuki and Nass, 1988; Yoshida *et al.*, 1998; Bertrand *et al.*, 2011).

Our results also indicate that although exposure to cold temperatures triggers the accumulation of proline in *Brachypodium*, the size of the proline pool of a given accession cannot be used to predict its freezing tolerance behaviour accurately since accessions with markedly different proline levels such as Bd29-1 and Bd1-1 exhibited very similar LT_50_ values (Figs 5, 6). This observation agrees well with the fact that no correlation could be established between variations in the proline pool during cold exposure and the level of freezing tolerance for arabidopsis and *Thellungiella* sp. (Lee *et al.*, 2012).

### Conclusions

This study demonstrates that cold acclimation and freezing tolerance mechanisms exist in *Brachypodium distachyon*. In response to LT, *Brachypodium* plants were able to accumulate *COR* gene transcripts and increase their pools of osmoprotectants. These changes are most probably responsible for the decrease in the LT_50_ values observed in all *Brachypodium* accessions tested and support our findings that cold acclimation modestly increases the freezing tolerance capacity of the *Brachypodium* model. Together with earlier findings that the *CBF* gene family is present in the genome of *Brachypodium* and that *BradiCBF1* is regulated by LT (Li *et al.*, 2012), the results presented here demonstrate that *Brachypodium* is able to acclimate to LT and to develop freezing tolerance.

This study also demonstrates that little to no natural genetic variation in terms of freezing tolerance exists among the *Brachypodium* diploid accessions tested and is the first to demonstrate that some *Brachypodium* accessions that do not necessitate a cold treatment in order to flower possess a facultative growth habit. We demonstrated that *Brachypodium* accessions Bd2-3, Bd3-1, Bd21 and Bd30-1 advanced to DR formation more rapidly and progressively reduced their FLN when exposed to cold for several days. Furthermore, a gradual accumulation of *BradiVRN1* transcripts and a synchronized decrease in the level of *COR* gene transcript were also observed in these accessions during cold acclimation.

Taken together, this study is the first to characterize the freezing tolerance capacities of the model plant *Brachypodium* and to demonstrate that the widely used Bd21 accession has a facultative growth habit.

## SUPPLEMENTARY DATA

Supplementary data are available online at www.oab.oxfordjournals.org and consist of the following. Figure S1: alignment of the deduced amino acid sequences of *Brachypodium distachyon* VRN1-related proteins. Figure S2: alignment of the deduced amino acid sequences of *Brachypodium distachyon* COR413 with related proteins.

Supplementary Data
