# Supplementary material for: Comparative analysis of the cold acclimation and freezing tolerance capacities of seven diploid Brachypodium distachyon accessions
Source: Ann Bot. 2013 Dec 8;113(4):681–93. doi: 10.1093/aob/mct283 (PMC3936580; doi:10.1093/aob/mct283)
Supplement: Supplementary Data [file supp_113_4_681__index.html]

Comparative analysis of the cold acclimation and freezing tolerance capacities of seven diploid Brachypodium distachyon accessions — Supplementary Data 

# Comparative analysis of the cold acclimation and freezing tolerance capacities of seven diploid *Brachypodium distachyon* accessions

## Supplementary Data

Supplementary Data

**Files in this Data Supplement:**

- Supplementary Data - Pdf file
