# Supplementary material for: Comparative analysis of the cold acclimation and freezing tolerance capacities of seven diploid Brachypodium distachyon accessions
Source: Ann Bot. 2013 Dec 8;113(4):681–93. doi: 10.1093/aob/mct283 (PMC3936580; doi:10.1093/aob/mct283)

SUPPLEMENTARY DATA

FIG. S1. Alignment of the deduced amino acid sequences of *Brachypodium distachyon* VRN1 related proteins. The alignment was generated by comparing the *Brachypodium* VRN1 sequence with the *Triticum monococcum* VRN1 (ACI24357.2) and *Triticum aestivum* VRT-1 (AAP33790.1) sequences. Identical and similar amino acids are shaded in black and grey, respectively. MADS-box domain, DNA-binding domain; I domain, intervening region; K domain, keratin-like domain; C domain, C terminal region; serine stretch, region rich in phosphorylation sites. Residues identified as being part of a nuclear targeting signal are indicated by *.

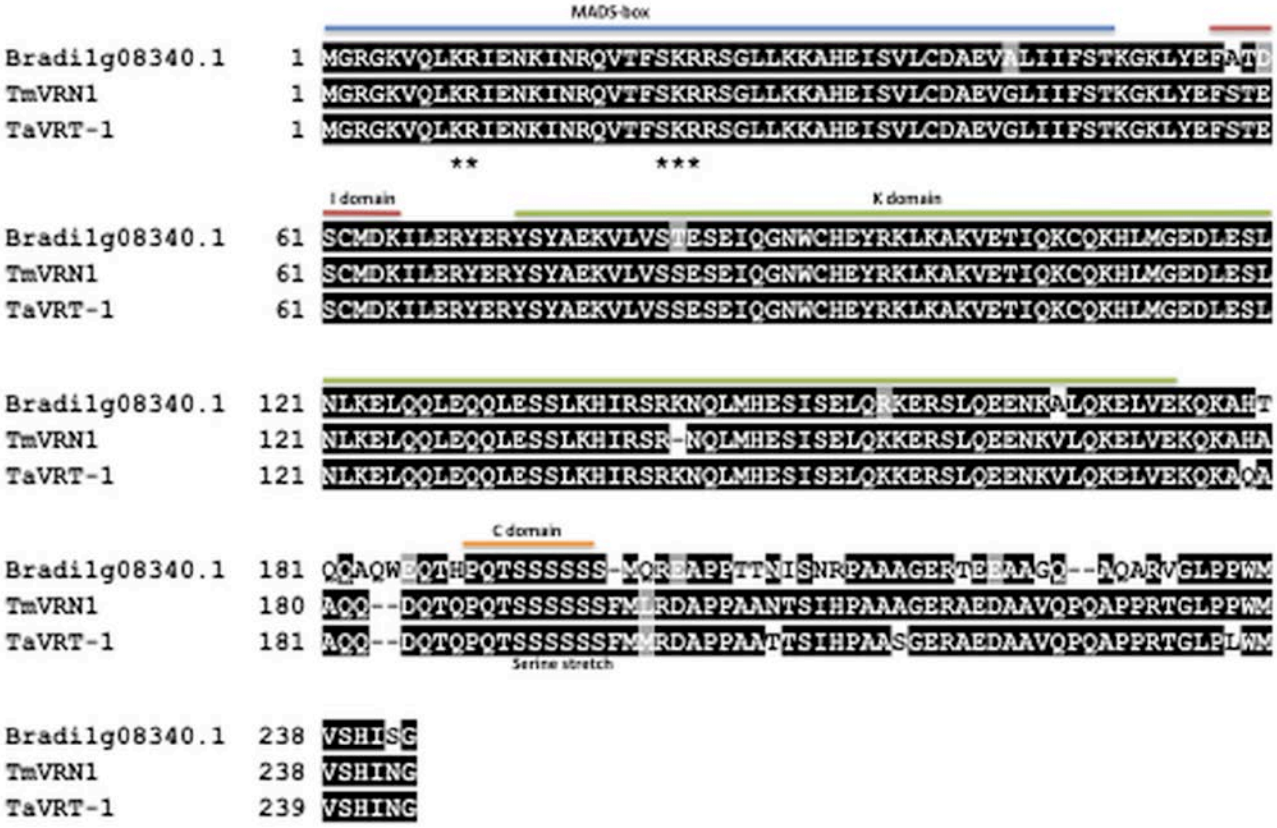

Fig S2. Alignment of the deduced amino acid sequences of *Brachypodium distachyon* COR413 with related proteins. The following protein sequences were used to generate the aligment: *Zea mays* COR413 (NM_001155133), *Oryza sativa* COR413 (AF283006.1), *Triticum aestivum* COR413 (U73216.1), *Arabidopsis thaliana* COR413 (NM_127156.3) and *hordeum vulgare* COR413 (AK356898.1). Identical and similar amino acids are shaded in black and grey, respectively.

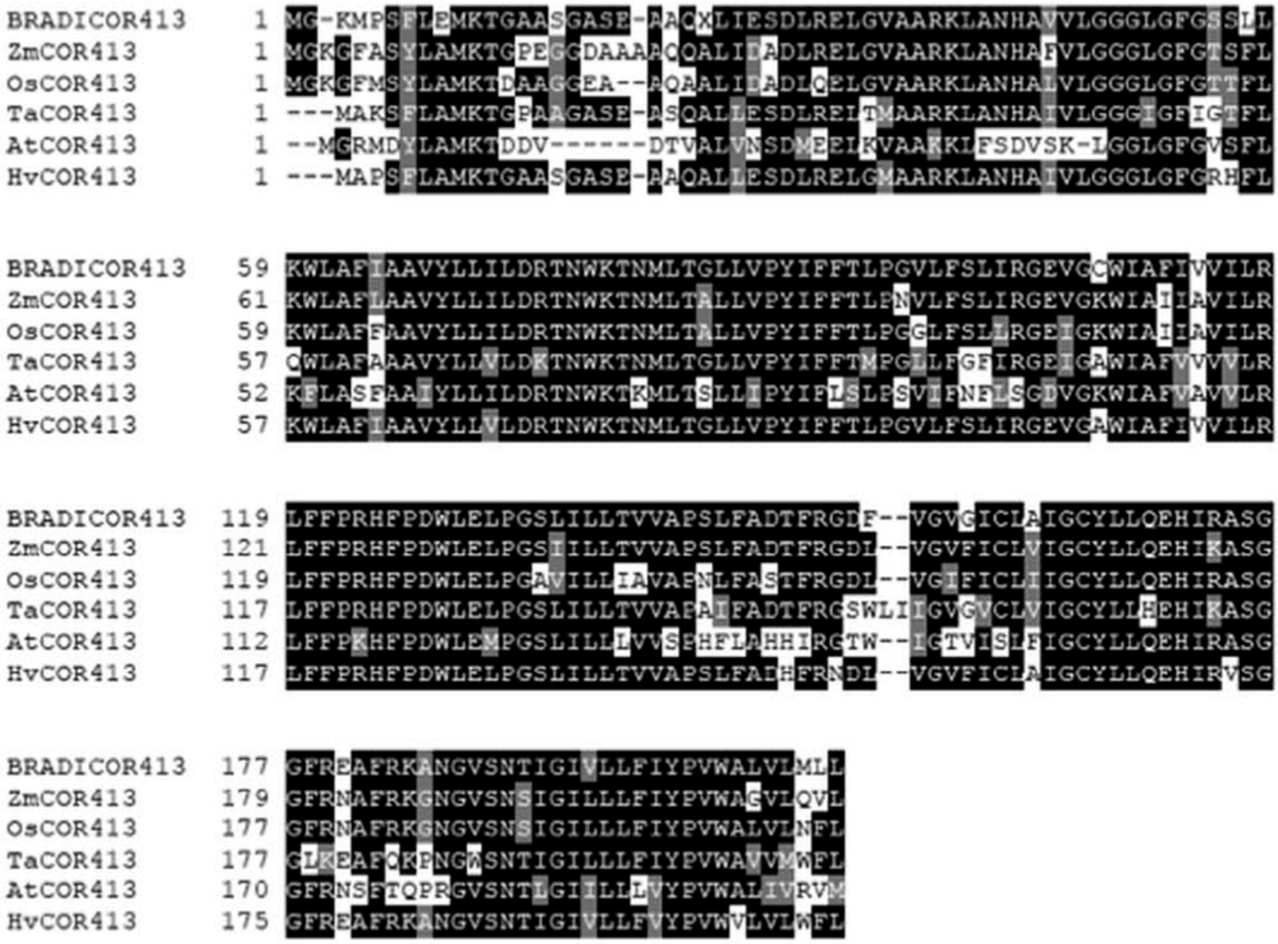

Supplement: Supplementary Data [file supp_mct283_mct283supp.pdf]
